# Developing context-sensitive, comprehensive newborn care protocols: integrating technologies with clinical care pathways for level 2 newborn units in Kenya

**DOI:** 10.7189/jogh.15.04271

**Published:** 2025-10-10

**Authors:** Grace Irimu, Edith Gicheha, Fareen Musa, Mary Waiyego, Dolphine Mochache, Audrey Chepkemoi, Felicitas Makokha, Allan Kayiza, Jacqueline Chesang, David Gathara, Jalemba Aluvaala, Roy Ndezwa, Maryanne Murugami, Joy Odhiambo, Rachael Kanguha, Brian Maugo, Caroline Mwangi, Aggrey Wasunna, Elizabeth Molyneux, Mike English, NBU-Protocols Development Group, Wairimu Kimani, Conrad Wanyama, Joyce Jebet, Abdullahi Hassan, Alex Macharia, Simon Kipkemoi, Simon Tamba

**Affiliations:** 1 Department of Paediatrics and Child Health, University of Nairobi, Nairobi, Kenya; 2 Health Services Unit, KEMRI-Wellcome Trust Research Programme, Nairobi, Kenya; 3 Rice360 Institute of Global Health Technologies, Rice University, Houston, Texas, USA; 4 The Nairobi Hospital, Nairobi, Kenya; 5 Kenyatta National Hospital, Nairobi, Kenya; 6 NEST360 Programme, Kenya; 7 Moi Teaching and Referral Hospital, Eldoret, Kenya; 8 Bungoma County Referral Hospital, Bungoma County, Kenya; 9 Department of Paediatrics and Child Health, Methodist University, Kenya; 10 Department of Public and Global Health, University of Nairobi, Nairobi, Kenya; 11 Defence Forces Memorial Hospital, Ministry of Defence, Kenya; 12 Longisa County Referral Hospital, Bomet County, Kenya; 13 Lumumba Sub-County Hospital, Kisumu County, Kenya; 14 Chuka County Referral Hospital, Tharaka Nithi County, Kenya; 15 Division of Neonatal and Child Health, Ministry of Health, Nairobi, Kenya; 16 Kamuzu University of Health Sciences, Blantyre, Malawi; 17 Nuffield Department of Medicine, University of Oxford, Oxford, UK

## Abstract

**Background:**

An estimated 2.3 million neonates die worldwide each year (47% of under-five mortality), with 75% occurring during the first week of life. The burden is highest in sub-Saharan Africa (n/N = 27/1000 neonatal mortality rate) and largely results from preventable conditions, such as prematurity, birth asphyxia, and infections. The Newborn Essential Solutions and Technologies (NEST360) programme supports health systems in resource-constrained settings (RCSs) through appropriate technologies, training, data use, and mentorship to reduce preventable neonatal deaths. The NEST360 programme, in partnership with the Kenya Ministry of Health, both inspired and enabled the development of evidence-based comprehensive newborn care protocols (NBU-Protocols) and a dissemination training. This article documents the development of the protocols and lessons learned to inform scalable solutions for RCSs.

**Methods:**

The NBU-Protocols and their dissemination programme were developed through a review of evidence on the care for small and sick newborns, followed by iterative feedback from stakeholders, including frontline health workers, academics, and researchers. The protocols were piloted and further revised following a national stakeholder workshop.

**Results:**

The NBU-Protocols comprise three chapters: clinical care pathways; standard operating procedures for NBU equipment; and step-by-step instructions for common clinical procedures performed in level 2 NBUs. The protocols were grounded in family-centred care and infection prevention and control principles. They were presented as e-protocols with hyperlinks, bookmarks, and cross-references to facilitate ease of use. The protocols dissemination programme, called Newborn ETAT+, was a three-pronged training approach taught by experts in the following groups: clinical care pathways by paediatricians; equipment parts and functions by biomedical engineers; and equipment use and care by NBU nurses. A third of the training was dedicated to interactive lectures, with the remainder focussed on demonstrations, simulations, clinical procedures on manikins, and hands-on experience with NBU equipment.

**Conclusions:**

The NBU-Protocols and training model highlight the potential of context-specific, multidisciplinary strategies to improve collaboration and standardise care in NBUs in low- and middle-income countries (LMICs).

Around 2.3 million newborns die worldwide each year, representing 47% of all under-five deaths [1]. Most of these deaths (75%) occur during the first week of life, with approximately one million newborns dying within the first 24 hours [2]. The United Nations Sustainable Development Goal (SDG) 3.2 aims to reduce the neonatal mortality rate (NMR) to less than 12 per 1000 live births by 2030 [3]. Sub-Saharan Africa (SSA) has the highest NMR in the world of 27 per 1000 live births, compared to the global average of 18 per 1000. Although it accounts for only 30% of global births, it contributes to 46% of all neonatal deaths [1].

The three leading causes of neonatal mortality in SSA are prematurity, intrapartum-related events (birth asphyxia/trauma) and infections, many of which are largely preventable with existing technologies [3–9]. Preventable comorbidities such as hypothermia and hypoxia significantly increase the risk of death among affected neonates [9–12]. Previously, in our scoping review, we identified modifiable gaps in newborn care: inadequate thermal/oxygen care, poor sepsis/jaundice management, and suboptimal resuscitation practices, which may contribute to poor outcomes in newborn units (NBUs) in low- and middle-income countries (LMICs) [13]. Achieving SDG 3.2 (NMR<12 per 1000 live births by 2030) requires empowering NBUs to provide standardised care for small and sick newborns (SSNBs) [4,5].

A handful of neonatal diseases/conditions account for over 80% of NBU admissions and more than 95% of deaths in Kenyan level 2 NBUs. They include intrapartum-related complications, respiratory distress syndrome, neonatal sepsis, neonatal jaundice, and uncomplicated low birth weight [14]. Despite these diseases being preventable and treatable, in Kenya’s referral hospitals, inpatient neonatal mortality in level 2 NBUs remains unacceptably high, exceeding 10% of all NBU admissions. The mortality rate among very low birth weight infants (1000–1499 g) in the NBUs of Kenya’s referral hospitals is over 40%, which is 10 times higher than in high-income countries [14]. Despite the increase in facility-based deliveries (88% nationally), many facilities lack essential lifesaving neonatal equipment for level 2 NBU care [15,16]. This is exacerbated by inadequate knowledge of the equipment's use and care, as well as a scarcity of healthcare providers (HCPs) explicitly trained in neonatal care [5].

To support LMICs in achieving the neonatal mortality rate SGD targets, the Newborn Essential Solutions and Technologies (NEST360) programme – an international alliance to end preventable deaths in African hospitals – was launched in 2019. It supports governments in strengthening health systems through four main approaches: introducing level 2 NBU technologies designed for LMICs contexts (**Box 1**); training HCPs on the use and care of the NBU technologies in the management of common neonatal conditions; improving the use of information for quality improvement; and mentorship [17].

Box 1 **The essential newborn care technologies provided by the NEST360 Programme at its introduction in Kenya**

NEST360 targeted WHO level 2 care newborn units and provided the following technologies to support care of small and sick newborns:

- Temperature stability (radiant warmer and temperature probe)
- Management of jaundice (light-emitting diode phototherapy lights and light meters)
- Respiratory support (oxygen concentrator and oxygen flow-rate splitters, continuous positive airway pressure machine, suction machine for newborns, pulse oximeter)
- Point of care diagnostics (glucometers appropriate for newborns and test strips)

First developed in 2005 and updated several times, the standards of care of the small and sick newborns (SSNBs) in level 2 NBUs in Kenya were previously derived from the Ministry of Health (MoH) basic paediatric protocols (BPPs) for children aged up to five years (including neonatal conditions). Recognising the need to update the BPP and procedural knowledge gaps among HCPs, Kenya’s MoH partnered with NEST360 to develop standalone newborn care protocols called ‘Comprehensive Newborn Care Protocols’ (NBU-Protocols) [18,19].

Here we aim to describe the process of developing the NBU-Protocols and the associated dissemination programme, and to share key lessons learned to inform similar efforts in other settings and guide future implementation.

## METHODS

We created the NBU-Protocols based on more than 20 years of experience using the BPPs for children up to five years of age [18,20–22]. Concurrently, we developed a dissemination programme for the NBU-Protocols, known as Newborn Emergency Triage, Assessment and Treatment plus Admission care (ETAT+), to enhance healthcare professionals' capacity to implement these protocols. This programme built on ETAT+, the training programme used for disseminating BPPs, referred to as Under5′s-ETAT+ [18].

### Development of the NBU-Protocols

We co-developed comprehensive newborn care protocols integrating technologies with clinical care appropriate for use in level 2 NBUs in Kenya and other LMICs.

### Scope of the NBU-Protocols

We tailored the scope of the NBU-Protocols using both experiences in newborn care provision in Kenya’s level 2 NBUs and the World Health Organization’s (WHO) recommendations for level 2 newborn care [4,5,14]. We developed evidence-based clinical care pathways (CCPs) to guide the management of common causes of morbidity and mortality in Kenya’s level 2 NBUs. To address the procedural knowledge gap, we developed and integrated step-by-step instructions for using and caring for essential NBU equipment and clinical procedures commonly performed on SSNBs, aligning them with CCPs.

### Development and iterative revision of the NBU-Protocols

The development of the NBU-Protocols involved a comprehensive literature search conducted through PubMed, Google Scholar, and available grey literature, focussing on evidence-based recommendations for the care of SSNBs that are accepted locally and internationally. A paediatrician (GI) and a neonatal nurse (EG) led the development of initial prototypes of the NBU-Protocols. The guidelines underwent iterative revisions based on feedback from multiple stakeholders, ensuring that the recommendations were context-sensitive and aligned with the needs of healthcare providers (HCPs) for delivering high-quality care to SSNBs in Level 2 NBUs. We presented the protocols to stakeholders using methods adapted from the Newborn ETAT+ training. Initially, the prototype protocols were shared with trainee paediatricians at the University of Nairobi (UoN), who also served as Under-5 ETAT+ trainers, as well as with Clinical Information Network (CIN) focal persons – paediatricians and nurses from the NBUs of 16 CIN hospitals. The CIN is a learning platform aimed at improving newborn and paediatric care through data utilisation and quality improvement in Kenyan referral hospitals [21]. We presented the revised protocols to the Kenya NEST360 Technical Group, which included three neonatologists, four neonatal nurses, one biomedical engineer, and two health system researchers, and revised them further based on the experts’ input. We then piloted the protocols in two NEST360 programme-implementing hospitals, and subsequently held a two-day workshop with neonatologists, paediatricians, neonatal nurses from Kenya county hospitals, academics, and policymakers from the MoH to gather further input and secure stakeholders’ buy-in (**Table 1**).

**Table 1:** Summary of the process of developing the NBU-Protocols and a programme for their dissemination (Newborn ETAT+)

Steps	Activities in developing the NBU-Protocols and ETAT+
1	Planning to update Basic Paediatric Protocols with guidance provided by MoH on how to integrate guidance on the use of newborn technologies with clinical care pathways.
2	Literature review, development of a prototype of NBU-Protocols and their dissemination programme termed as Newborn ETAT+.
3	Testing of the prototype NBU-Protocols and a dissemination programme among University of Nairobi Under5′s-ETAT+ trainers, frontline NBU HCPs and the Kenya NEST360 Technical Group.
4	Two pilot training sessions were conducted in hospitals implementing the NEST360 programme. Revised materials were presented to neonatologists and paediatricians in academia.
5	MOH-led validation of the NBU-Protocols and further refinement.
6	Development of e-protocols that included hyperlinks, bookmarks and cross-references.

ETAT+ *–* Emergency Triage Assessment and Treatment Plus admission care, HCPs – healthcare providers, MoH – Ministry of Health, NEST – Newborn Essential Solutions and Technologies, NBU – newborn unit, NBU-Protocols – Comprehensive Newborn Care Protocols-Integrating Technologies with Clinical Care

### Validation of the NBU-Protocols by stakeholders

The NBU-Protocols were validated through a three-day virtual meeting involving a multidisciplinary panel of stakeholders, including representatives from the MoH, development partners, policymakers, academics, and county health officials from all 47 Kenyan counties. The panel comprised eight neonatologists, 23 paediatricians, nine neonatal nurses, 28 NBU nurses, five clinical officers, 10 technology focal persons from the NEST360 programme, and two members of the global NEST360 team. Half of the participants were Newborn ETAT+ trainers with extensive experience in evidence-based NBU care. The validation aimed to ensure the protocols were acceptable, feasible, usable, and applicable across diverse settings. Led by the MoH, the process followed national guidelines for clinical protocol validation, and included structured discussions to address contentious areas through consensus, supported by scientific evidence and local expertise. Panellists recommended publishing the protocols electronically to allow timely updates. The MOH officially endorsed and launched the protocols in November 2022 (**Table 1**).

### Development of newborn ETAT+ course – a dissemination programme for NBU-Protocols

We developed Newborn ETAT+ as a structured training programme to ensure the NBU-Protocols were both teachable and implementable in resource-constrained settings. It drew on the conceptual framework of the Under-5 ETAT+ training, refining it to address the specific needs of newborn care and overcome challenges previously encountered during national scale-up efforts of the original program [18–20,22,23].

We aligned the curriculum with the chapters and scientific foundations of the NBU-Protocols. Two researchers (GI, EG) led the development, supported by experienced Under-5 ETAT+ trainers. We designed a modular programme that delivered one disease entity or condition at a time, allowing for a cumulative learning experience. Each module had clearly defined learning objectives and outcomes.

For theoretical instructions, we developed PowerPoint slides to present evidence-based prenatal and postnatal care, explain the rationale and structure of the CCPs, and walk participants through the step-by-step standard operating procedures (SOPs) and clinical procedures. These slides also included guidance on monitoring for treatment complications and preventing adverse outcomes. We provided references at the bottom of each slide to ensure scientific rigor and transparency.

We standardised practical sessions through the development of an instructor’s manual. This manual, aligned with the course objectives, guided instructors in delivering hands-on training using SOPs for the use and care of NBU equipment, and clinical procedures outlined in the NBU-Protocols. It also included responsive clinical scenarios for simulation, focussed on key skills such as equipment assembly, patient monitoring, and procedural competence. We adapted Peyton’s four-step approach (demonstration, deconstruction, comprehension, and execution) to teach these skills effectively [24].

We integrated simulations into each module, with structured debriefing sessions to foster reflective learning. We encouraged participants to examine their assumptions, unlearn unsafe practices, and evaluate the feasibility of adopting the protocols in their settings. These discussions promoted behaviour change and clinical reasoning [25].

A team of academic experts (GI, EG, BM, AW) developed and reviewed assessment tools, including written exams and practical evaluations with answer keys and marking schemes. We conducted two pilot five-day trainings in hospitals implementing the NEST360 programme, and we used feedback to refine the contents and delivery methods.

The development of Newborn ETAT+ was guided by educational theories and adult learning principles aimed at promoting competence, confidence, and behaviour change in clinical practice. This approach ensured the course was contextually appropriate, sustainable, and scalable for implementation across diverse healthcare settings in Kenya (**Table 2**).

**Table 2:** Application of theories in the development of Newborn ETAT+

Lessons learnt from previous work	Theory applied	Application of theories in the development of Newborn ETAT+
A shift in organisational culture and leadership that positively influences the micro-system needs to be re-engineered from within [23].	Normative- re-educative approach [26]	We envisaged that the approach would create an enabling environment and commitment to embracing hospital norms and culture that support the adoption of evidence-based care for SSNBs by building the capacity of HCPs in self-directed learning.
Minimising hierarchy and rigid boundaries strengthens clinical teams by fostering a shared professional identity [19,23].	System management theory [27]	The interdependency of transdisciplinary teams was emphasised to bring out the synergism of the skills of different cadres in caring for the SSNBs. This was achieved by reaffirming the identity of each cadre with role clarity while recognising the fluid boundaries of the different disciplines. HCPs were encouraged to collaborate and draw on the team's knowledge.
Inadequacies in self-regulation [19].	Self-regulation theory [28]	The NBU-Protocols and Newborn ETAT+ training included newborn care standards that participants were required to reflect on. These standards were used as audit criteria to monitor the adoption of the protocols.
Health workers maintain their primacy in the care of patients and protect their profession [19].	Family Centred Care Theory [29]	The principles of family-centred care underpin the entire course and the NBU-Protocols.
Inadequate attention to transition and continuum of care between HCPs and across working shifts/handover processes [30,31].	Swiss Cheese model [32]	Emphasis was on the importance of monitoring patients and interpreting clinical and laboratory findings to recognise early signs of worsening conditions, as well as timely communication of these findings between team members and across relevant disciplines.
Insufficient self-driven reading culture on quality-of-care issues [19,22,33].	Self-directed learning and adult learning theories [34]	A WhatsApp group was created for all participants. Pre-course reading materials were distributed via email and WhatsApp three weeks before the training. One week prior, participants discussed video topics, including newborn resuscitation, danger signs in sick and small newborns (SSNBs), breast milk expression, and heel prick procedures [35]. Trainers received technical reference materials for the Newborn ETAT+ training, emphasising the importance of these resources during facilitator rehearsals one week before the training.
HCPs may have inadequate procedural knowledge that affects the performance of clinical skills [19,22].	Cognitive Learning Theory and Behaviourist learning theory [36]	We modified Peyton's Four-Step-Approach, which consists of demonstration, deconstruction, comprehension, and execution for clinical procedures [24]. Simulations and group discussions allowed participants to reflect on their assumptions and how various internal and external factors may have influenced their practices.
A credible training team positively influences internal working routines, commitments or capabilities (micro-system). HCPs are happy to consult trusted, knowledgeable individuals [23].	Social Influence Theory [37]	The Newborn ETAT+ trainers were experienced focal persons from hospitals equipped with NEST360 technologies. GI and ME, key contributors to Kenya’s successful BPPs, trained many paediatricians [18,38]. EG, a leader in neonatal nursing education, mobilised NBU nurses, while AW, regarded as the father of neonatology in Kenya, provided significant leadership and expertise.

ETAT+ – Emergency triage, assessment and treatment plus admission care, HCPs – healthcare providers, NBU – newborn unit, SSNBs – small and sick newborns

## RESULTS

This work produced two primary outputs: the NBU-Protocols, which integrate essential technologies with clinical care in Level 2 NBUs, and the Newborn ETAT+ training programme to build healthcare workers’ capacity to implement these protocols in routine practice.

### Comprehensive NBU-Protocols – integrating technologies with clinical care

The validated NBU-Protocols consisted of an introduction to newborn care quality statements, followed by three chapters: CCPs; SOPs for level 2 NBU equipment (the content was limited to commercially available equipment); and step-by-step instructions for common clinical procedures performed in level 2 NBUs.

The quality statements comprised nine standards for improving the quality of care for SSNBs, each incorporating sub-statements adapted from the WHO quality-of-care framework [39]. We developed the ninth standard, ‘all essential newborn technologies are used and maintained per MoH protocols/manufacturer’s instructions to ensure they are safe for use’, along with six associated quality sub-statements [40].

The NBU-Protocols targeted clinicians and nurses who care for SSNBs, focussing on illness-specific care of the common serious neonatal conditions that occur in the first week of life in Kenyan level 2 NBUs. The protocols followed evidence-based recommendations accepted both locally and internationally; for example, we adapated the newborn resuscitation guidelines from the American Heart Association guidelines [41] and neonatal jaundice nomograms from the Queensland Clinical Guidelines [42]. We adapted the procedures for using the equipment provided by NEST360 from SOPs developed by an international NEST360 team with expertise in medical education and experience working in LMICs [43].

We emphasised cross-cutting forms of supportive care enabled by better equipment use for early detection, treatment and monitoring of hypoxaemia, hypothermia and hypoglycaemia throughout the NBU-Protocols, which were grounded in the principles of family-centred care (FCC) and infection prevention and control (IPC). We presented the protocols in the electronic form with hyperlinks, bookmarks, and cross-references to enhance their structure, organisation, navigation, and functionality (**Table 3**) [40].

**Table 3:** Chapters and contents of the NBU-Protocols

Introduction: newborn care quality statements	Chapter 1: illnesses/conditions whose clinical care pathways were provided	Chapter 2: NBU equipment whose standard operating procedures were provided	Chapter 3: common clinical procedures whose step-by-step instructions were provided
Nine standards for improving the quality of care for small and sick newborns; quality statements for each of the standards consistent with the WHO quality of care framework	Newborn resuscitation; birth and cord-cutting; breastfeeding techniques; prevention and treatment of hypoglycaemia; feeds and fluids; supporting respiratory efforts using conventional oxygen therapy and CPAP; neonatal seizures; assessment and treatment for possible bacterial infection; assessing the severity of jaundice and using hour-specific nomograms for administering phototherapy	Radiant warmers; neonatal suction machines; CPAP; oxygen concentrators; oxygen flowrate splitters; pulse oximeters; light emitting diode phototherapy lights; glucometers	Newborn resuscitation; use of plastic wraps for preterm newborns; administering oxygen; correct breastfeeding techniques; expressing breast milk; cup feeding a newborn; inserting an oral/nasal gastric tube; administering buccal glucose; performing heel pricks; inserting intravenous access

CPAP – continuous positive airway pressure, NBU – newborn unit, WHO – World Health Organization

### Newborn ETAT+ training programme

Newborn ETAT+ was a five-day physical training programme conducted in the health facilities to enhance healthcare workers’ capacity to utilise NBU-Protocols in their routine clinical practice.

The training sessions aimed to improve participants' knowledge and competencies in assessing and managing newborns with conditions such as intrapartum-related complications, jaundice, sepsis, and low birth weight or prematurity, including the provision of appropriate supportive care, all grounded in the principles of FCC and IPC. The training was also underpinned by adult learning principles, encouraging active engagement from learners. It consisted of interactive instructor-led plenary sessions to impart theoretical knowledge and participant-led practical sessions to support better care for the SSNBs as per the NBU-Protocols.

The plenary sessions consisted of interactive lectures that focussed on the application of the CCPs and demonstrated the use of SOPs for NBU equipment and clinical procedures described in the NBU-Protocols. Newborn ETAT+ plenary sessions followed a three-pronged format: CCPs taught by paediatricians; parts, functions, use, and care of the equipment, taught by biomedical engineers or equipment nurses; and clinical procedures and monitoring of the SSNBs were taught by NBU nurses. PowerPoint presentations for the plenary sessions are available on the UoN website [44]. We dedicated one-third of the Newborn ETAT+ training time to theory sessions, while we spent the remainder in plenary sessions focussed on demonstrations of care, equipment use, and clinical procedures, along with small-group practical sessions for hands-on experience.

The corresponding practical session followed the plenary sessions in small groups of six to eight participants. Two Newborn ETAT+ instructors – a paediatrician and a nurse – supervised and guided each group according to the Newborn ETAT+ instructor’s manual. The participants practised what was taught in the plenary session, following the step-by-step instructions described in the SOPs in the NBU-Protocols. We conducted structured role clarification sessions to delineate responsibilities and highlight the interdependence of tasks among team members. The instructors conducted formative assessments by observing the participants' behaviour during the simulations and discussions during the post-simulation debriefing (Table S1 in the **Online Supplementary Document**) [45].

We used a structured Newborn ETAT+ Instructor's Manual to guide the practical sessions, providing detailed instructions for teaching clinical skills, conducting simulations, facilitating group discussions, and leading role plays. The manual incorporated the use of NBU equipment and consumables, procedure-specific baby dolls or manikins, and breast models to support hands-on learning and scenario-based practice. The manual offered structured guidance for assessing both individual and team-based tasks within the simulation exercises. It introduced a standardised framework for post-simulation debriefing sessions aimed at enhancing clinical reasoning and reflective thinking, as well as identifying performance gaps and systemic deficiencies. Moreover, the manual provided instructors with strategies for implementing learner-centred teaching approaches, thereby fostering a safe and supportive learning environment conducive to active engagement and deeper learning [45,46]. Additionally, it guided participants in integrating the newly developed electronic NBU-Protocols into their smartphones' home screens and navigating through these e-protocols, facilitating easier access and utilisation during clinical practice.

The written examination was a recall-plus application multiple-choice examination with four options, one of which was the correct answer [47]. We recommended a retake of the entire Newborn ETAT+ for those scoring below 50%. The practical examination comprised simulated, participant-led clinical scenarios designed to test the application of CCPs, the correct use of NBU equipment and performance of clinical procedures using manikins and essential equipment, underpinned by FCC and IPC measures. Each candidate underwent two 10-minute simulation exercises, with each station staffed by two assessors who independently evaluated performance using a standardised checklist. The checklist outlined critical steps that, if not performed, would compromise the patient's outcome. Candidates who missed performing any critical step failed the practical and either re-sat the practical examination after a remedial session or retook the whole five-day Newborn ETAT+ course as determined by pre-defined criteria. Those who had flawless performances scored excellent grades.

Following the training, we installed NEST360 equipment in the health facilities NBU (**Box 1**). Two nurses who were Newborn ETAT+ instructors provided an additional two-day mentorship at the facility. This mentorship addressed implementation challenges, supported service reorganization to optimize NEST360 clinical bundles, and promoted adopting new NBU care standards and implementing the NBU-Protocols. Table S2 in the **Online Supplementary Document** summarises the CCPs with corresponding key SOPs for the NBU equipment and clinical procedures commonly performed in Level 2 NBUs taught in Newborn ETAT+.

### Lessons learned

Stakeholder engagement was central to the iterative development and refinement of the NBU-Protocols and the Newborn ETAT+ training programme, yielding several key lessons.

### Enhancing teamwork and identity through co-development and multidisciplinary training

The entire process for development of NBU-Protocols was a collaborative effort of the MoH, professional bodies, academia, non-governmental organisations, NBU nurses, paediatricians, and medical engineers. This inclusive process fostered ownership and accountability across stakeholders. Engaging multidisciplinary teams in facilitating the three-pronged Newborn ETAT+ enhanced teamwork, cohesion, and cadre-specific identity. Nurse-led teams developed SOPs for each piece of NBU equipment and the corresponding clinical procedures. Nurses leading sessions related to their daily tasks were inspired to perfect their teaching, gaining a clear identity and respect within the NBU. The collaboration also improved healthcare professionals' understanding of the critical role of biomedical engineers in the NBUs. We observed that traditional professional hierarchies could be overcome by prioritising clinical expertise over professional titles or seniority, fostering an environment of mutual respect, mitigating ‘silo mentality’, encouraging multidisciplinary care, and minimising role conflicts [48]. The use of generic training materials, hospital teams, and UoN Under5′s ETAT+ trainers fostered shared ownership of the NBU-Protocols and enabled their integration into pre-service curricula and routine practice.

### Leveraging established protocols for broader acceptance of the NBU-Protocols

The structure and format of the NBU-Protocols and Newborn ETAT+ closely mirrored the BPPs and Under-5 ETAT+ – programmes that have been widely implemented in Kenya since 2005 [14,18,31,38]. This alignment facilitated rapid uptake and acceptance among healthcare providers and institutions. Many of the Newborn ETAT+ instructors were also experienced Under-5 ETAT+ trainers, ensuring continuity and consistency in teaching methods. The MoH-led validation ensured the protocols were generic and not limited to specific brands of equipment supplied by NEST360, further enhancing acceptance and scalability at both in-service and pre-service institutions.

### Digital transformation of the NBU-Protocols

Although initially unplanned, the evolution of the protocols into a digital format was driven by stakeholder recommendations and the need for adaptability. The e-protocols allowed for ongoing updates, incorporating new WHO guidelines and recent evidence [4]. This adaptability increased usability and ensured protocols remained current and relevant.

### Suboptimal normative practices compounded a weak culture of self-directed learning

Stakeholders' engagement revealed that many prevailing clinical practices lacked evidence-based foundations. Limited self-directed learning – such as not reading equipment manuals or keeping up with clinical guidelines – contributed to improper equipment use and suboptimal care. During the interactive sessions, it became evident that many HCPs were unaware of emerging technologies, high-impact interventions, and effective data use in guiding care. Training approaches must recognise the pre-existing knowledge – both beneficial and harmful – that care providers bring. Safe learning environments should support reflection, unlearning of harmful habits, and integration of best practices into daily care (Table S3 in the **Online Supplementary Document**) [35].

## DISCUSSION

We describe a novel, multidisciplinary initiative designed to enhance care in level 2 NBUs in resource-constrained settings. The NBU-Protocols integrate structured CCPs, equipment-specific SOPs, and practical hands-on clinical procedures, providing context-adapted tools for resource-constrained settings. The three-pronged dissemination strategy – CCPs delivered by paediatricians or neonatologists, SOPs by biomedical engineers, and clinical procedures by nurses – redefined the roles and identity of NBU staff, particularly nurses and biomedical engineers, enhancing inter-professional collaboration and teamwork.

The development and implementation of the NBU-Protocols and Newborn ETAT+ revealed significant knowledge gaps among healthcare providers. These gaps reflect pre-service and in-service training deficiencies, which our protocols aimed to address [19,49].

While the absence of essential NBU equipment remains a documented barrier in LMICs, our findings highlight an additional challenge: limited understanding of equipment specifications and improper clinical procedures, which often result in suboptimal use of available technologies [50]. These gaps underscore the urgent need for full implementation of the MoH’s training policy to support continuous professional development and promote competency-based service delivery [51]. Addressing these issues also requires reforms in pre-service education to nurture a culture of self-directed learning, while healthcare leadership must foster an environment that supports ongoing learning and the timely adoption of new technologies.

Our approach underscores the complexity of clinical care for SSNBs, highlighting the need for robust multidisciplinary teams. Training acknowledged participants' existing expertise while fostering reflective dialogue, hands-on learning and safe practice change. Structured debriefing processes further promoted understanding, enhanced learning, and contributed to improved clinical competence [45,46]. Formative and summative simulation-based assessments, aligned with best practices, also helped strengthen clinical competence [52]. Additionally, protocol development benefited from feedback across disciplines and alignment with national training programmes [18].

However, we observed several limitations. The protocols were not integrated into electronic systems, limiting real-time usability [52]. Newborn ETAT+ relied heavily on in-person delivery, which is less scalable in high-turnover, low-resource settings due to the absence of asynchronous or online components [49,53,54]. Lastly, to sustain practice change, protocols and training must be embedded within broader health systems and supported by strong leadership, organisational readiness, and community-based approaches. Without these, improvements in SSNB care may not be fully realised [49,54–56].

## CONCLUSIONS

The NBU-Protocols and associated training model demonstrate that context-specific, multidisciplinary approaches can enhance collaboration and promote practice alignment among providers in NBUs in LMICs. While competence improvements were not directly measured, the model offers a foundation for strengthening provider engagement and standardising care. To sustain impact, future efforts should prioritise digital integration, flexible training platforms, and alignment with broader health system strengthening initiatives. Building resilient systems that empower providers and support continuous learning remains essential for advancing newborn care in resource-limited settings.

## Additional material


Online Supplementary Document
