# Supplementary material for: Developing context-sensitive, comprehensive newborn care protocols: integrating technologies with clinical care pathways for level 2 newborn units in Kenya
Source: J Glob Health. 2025 Oct 10;15:04271. doi: 10.7189/jogh.15.04271 (PMC12512004; doi:10.7189/jogh.15.04271)
Supplement: Online Supplementary Document [file jogh-15-04271-s001.pdf]

Supplement to: Irimu G, Gicheha E, Musa F, Waiyego M, Mochache D, Chepkemoi A, Makokha F, Kayiza A, Chesang J, Gathara D, Aluvaala J, Ndezwa R, Murugami M, Odhiambo J, Kanguha R, Maugo B, Mwangi C, Wasunna A, Molyneux E, English M; NBU-Protocols Development Group. Developing context-sensitive, comprehensive newborn care protocols: integrating technologies with clinical care pathways for level 2 newborn units in Kenya. J Glob Health, 2025;15:04271.

Supplemental Table S1: The Newborn ETAT+ Provider Course timetable

Newborn **ETAT+ Provider Course Timetable. Dates:________________Hospital Name**:

Time	Activity	Venue	Facilitated by
**Day 1 Date……**			
8.45 - 9.00am	Registration	Plenary	
9.00 - 9.30am	Introductions/Climate setting	Plenary	
9.30 - 9.50am	Introduction to NEST360 Programme	Lecture	
9.50 - 10.30am	Introduction to Comprehensive newborn Protocol and standards of Care	Lecture/discussion	
**10.30 - 11.00am**	**Break**		
11.00 - 11.30am	Infection Prevention & Control (IPC)	Lecture/discussion	
11.30 - 12.00noon	Family Centered Care (FCC) & Pain Management	Lecture/discussion	
12.00noon - 1.00pm	Newborn Transition and Adaptation	Lecture/discussion	
**1.00pm - 2.00pm**	**Lunch**		
2.00 - 4.00pm	Newborn Clinical Signs and Symptoms	Lecture, Video & Discussion	
**4.00pm**	**Closure**		
**Day 2 Date:**			
8.00am - 8.15am	Registration	Plenary	
8.15am - 8.30am	Recap/Pending issues	Plenary	

8.30am - 10.30am	Newborn Resuscitation (NR) & Equipment use	Interactive Lecture, Skill teaching & Demo	
**10.30am - 11.00am**	**Break**		
11.00am - 1.00pm	Practical Sessions - NR and Use of radiant warmer & Suction Machine	Groups	
**1.00pm - 2.00pm**	**Lunch**		
2.00pm - 3.30pm	Feeds and Fluids Management Including breast milk expression and use	Interactive Lecture, demonstrations and exercises	
3.30pm - 5.00pm	Practical Sessions - NG/OG insertion, expression, cup feeding, drills, breastfeeding techniques	Groups	
**5.00pm**	**Closure**		
**Day3 Date:**			
8.00am - 8.15am	Registration	Plenary	
8.15am - 8.30am	Recap/Pending issues	Plenary	
8.30am - 10.30am	Pulse oximetry, Oxygen Therapy and monitoring	Interactive	
**10.30am - 11.00am**	**Break**		
11.00am - 1.00pm	RDS and Use of CPAP	Interactive Lecture	
**1.00pm - 2.00pm**	**Lunch**		
2.00pm - 5.00pm	Practical sessions - CPAP & Oxygen therapy	Groups	
**5.00pm**	**Closure**		

**Day 4 Date:**

8.00am - 8.15am	Registration	Plenary	
8.15am - 8.30am	Recap/Pending issues	Plenary	
8.30am - 9.30am	Neonatal hypoglycemia	Interactive lecture	
9.30am - 10.30am	Neonatal Seizures	Interactive Lecture	
**10.30am - 11.00am**	**Break**		
11.00am - 1.00pm	Neonatal Jaundice	Interactive Lecture	
**1.00 - 2.00pm**	**Lunch**		
2.00pm - 5.00pm	Practical sessions - Hypoglycemia and Phototherapy	Groups	
**5.00pm**	**Closure**		
**Day 5 Date :**			
8.00am - 8.15am	Registration	Plenary	
8.15am - 8.30am	Recap/Pending issues	Plenary	
8.30am - 10.30am	Mixed Scenarios	Groups/all stations	
**10.30am - 11.00am**	**Break**		
11.00am - 12.00pm	Mixed Scenarios	Groups/all stations	
12.00pm - 1.00pm	Practical Assessment	Interactive Lecture	
**1.00 - 2.00pm**	**Lunch**		
2.00pm - 4.00pm	Practical Assessment/Post Test/Evaluation	Groups/all stations	
4.00pm - 4.30pm	Certification and Closing Remarks	Plenary	

Supplemental Table S2: Clinical care pathways and key standard operating procedures for the equipment and clinical procedures in the NBU-Protocols and Newborn ETAT+

Clinical care pathways	Key standard operating procedures covered in the NBU-Protocols and Newborn ETAT+ corresponding to the CCP	*Commonly performed clinical procedures for the corresponding CCP whose guidance was provided in the NBU-Protocols and Newborn ETAT+

Newborn resuscitation and maintenance of warmth	• Testing the functionality of a radiant warmer<br>• Use of radiant warmer, a temperature probe, timers and setting alarms<br>• Testing functionality and use of the suction machine, setting suction pressures	• Newborn resuscitation<br>• Use of polythene wrap and radiant warmer for maintaining a warm chain after birth<br>• Clearing the airway using a suction machine and suction catheter
Prevention and management of early hypoglycaemia	• Use of newborn glucometer<br>• Conducting quality control test	• Heel prick<br>• Application of buccal glucose
Feeding the small and sick newborns	• No NBU equipment taught.	• Breastfeeding techniques using breast models and manikins<br>• Hand expression of breast milk using breast models<br>• Cup feeding technique<br>• Inserting and confirming the position of the oral or nasal gastric tube<br>• Using dosage charts for oral and intravenous feeds and fluids
Oxygen therapy in newborns	• Use of oxygen concentrator<br>• Use of oxygen flow rate splitter<br>• Use of pulse oximeter and securing the probe on the newborn	• Fixing and securing the nasal prongs and testing their functionality<br>• Titrating oxygen therapy to target oxygen saturation.<br>• Weaning off and stopping oxygen therapy
Respiratory distress syndrome	• Preparing CPAP for use<br>• Testing functionality of CPAP	• Inserting and confirming the position of the oral gastric tube<br>• Preparing baby for CPAP, sizing the CPAP prongs and preparing the interface<br>• Titrating and stopping CPAP
Neonatal sepsis<br>Neonatal seizures	• No equipment taught	• Fixing and securing peripheral intravenous lines
Early detection and treatment of neonatal jaundice	• Preparing light-emitting diode phototherapy lights<br>• Testing functionality of phototherapy lights using the light meter	• Preparing neonate to receive phototherapy<br>• Reading the neonatal jaundice nomograms and providing correct irradiance<br>• Monitoring a neonate receiving phototherapy

CCPs- Clinical care pathways; CPAP – continuous positive airway pressure; ETAT+ - Emergency triage assessment and treatment Plus admission care; NBU – Newborn Unit; SOPs – standard operating procedures;
**Underpinned by principles of family-catered/ centred care and infection prevention and control for each intervention.*

Supplemental Table S3: Suboptimal normative practices that were exacerbated by a poor self-driven reading culture among health care providers

Topic	Suboptimal practices of health care providers that formed the basis of discussions during the stakeholder's engagement
Infection Prevention and Control	Hand hygiene practices were suboptimal, with healthcare professionals infrequently washing hands between patients. Misunderstandings regarding using detergents and disinfectants led to improper infection control practices, including the misuse of enzymatic detergents as disinfectants, alcohol-based solutions not allowed sufficient time to dry and incorrect dilution and storage of sodium hypochlorite. Neonates often shared incubators, bassinets, and cots, with multiple infants placed in a single bassinet, limiting opportunities for proper cleaning and increasing the risk of hospital-acquired infections.
Family-centred care	Caregivers were allowed entry into NBUs strictly for three-hourly feeds, and health workers rarely explained the disease, treatment plans and outcomes, all of which were attributed to understaffing. Kangaroo care and rooming-in were rarely practiced correctly, it was started/stated
Newborn resuscitation	After cord clamping, newborns were typically placed on radiant warmers without skin-to-skin contact during the first hour after birth, even when assisted ventilation was not needed. The radiant warmer's control panel, temperature probes, and 'baby mode' setting were rarely used. Additionally, plastic wraps to prevent heat loss in preterm infants were infrequently applied and, when used, were placed after drying the infant.
Feeds and fluids	Preference to calculate the feeds using mobile phone calculators rather than checking in feed charts in the Basic Paediatric Protocols. Routine gastric lavage was the norm before gastric feeds. Trophic feeds for newborns on intravenous fluid were rarely given. Feeding stable babies within the first one hour after birth was a major challenge because of the perception of 'no breastmilk'.
Prevention and treatment of early hypoglycaemia	Glucose testing in newborns was frequently conducted using unsafe sites such as fingers, toes, and the middle of the heel. Oral gel for hypoglycaemia management was not in use. Stakeholders recommended application of 50% dextrose on the buccal mucosa to treat hypoglycaemia, given the unavailability of 40% dextrose gel in Kenya.
Oxygen delivery	Oxygen therapy for neonates predominantly involved adult or paediatric face masks with non-rebreathers, as neonatal nasal prongs were unavailable. Paediatric nasal prongs, when used, were cleaned and reused. Clinical signs primarily guided oxygen therapy, with intermittent oxygen saturation monitoring being infrequent. Oxygen concentrators were employed for emergencies with inadequate time to concentrate oxygen. Routine oxygen concentration analysis was seldom performed in hospital plants, outlets, and concentrators.
CPAP	Few facilities had CPAP machines, and where they were available, using them was reported to be challenging
Neonatal suction machine	Use of an adult suction machine and not setting suction pressures was the norm in all NBUs.<br>Nasogastric tubes were used for suction instead of suction tubes. Poor technique of suctioning using a suction machine
Phototherapy lights	Nomograms, light meters, and control panels for adjusting phototherapy intensity were not used, and phototherapy prescriptions did not account for irradiance. Treatment was ineffective due to multiple newborns being placed under a single

	phototherapy unit, resulting in frequent referrals for exchange transfusions. However, the need for exchange transfusion significantly decreased after implementing NEST technologies and staff training on NBU-Protocols.

CPAP – continuous positive airway pressure; ETAT+ - Emergency Triage, Assessment and Treatment plus admission care;

HCP- Health Care Provider, NBU- Newborn Unit, NEST- Newborn Essential Technologies and Solutions
